# Screening and predicted value of potential biomarkers for breast cancer using bioinformatics analysis

**DOI:** 10.1038/s41598-021-00268-9

**Published:** 2021-10-21

**Authors:** Xiaoyu Zeng, Gaoli Shi, Qiankun He, Pingping Zhu

**Affiliations:** School of Life Sciences, Zhengzhou University, Zhengzhou, China

**Keywords:** Breast cancer, Cancer screening

## Abstract

Breast cancer is the most common cancer and the leading cause of cancer-related deaths in women. Increasing molecular targets have been discovered for breast cancer prognosis and therapy. However, there is still an urgent need to identify new biomarkers. Therefore, we evaluated biomarkers that may aid the diagnosis and treatment of breast cancer. We searched three mRNA microarray datasets (GSE134359, GSE31448 and GSE42568) and identified differentially expressed genes (DEGs) by comparing tumor and non-tumor tissues using GEO2R. Functional and pathway enrichment analyses of the DEGs were performed using the DAVID database. The protein–protein interaction (PPI) network was plotted with STRING and visualized using Cytoscape. Module analysis of the PPI network was done using MCODE. The associations between the identified genes and overall survival (OS) were analyzed using an online Kaplan–Meier tool. The redundancy analysis was conducted by DepMap. Finally, we verified the screened HUB gene at the protein level. A total of 268 DEGs were identified, which were mostly enriched in cell division, cell proliferation, and signal transduction. The PPI network comprised 236 nodes and 2132 edges. Two significant modules were identified in the PPI network. Elevated expression of the genes Discs large-associated protein 5 (*DLGAP5*), aurora kinase A (*AURKA*), ubiquitin-conjugating enzyme E2 C (*UBE2C*), ribonucleotide reductase regulatory subunit M2(*RRM2*), kinesin family member 23(*KIF23*), kinesin family member 11(*KIF11*), non-structural maintenance of chromosome condensin 1 complex subunit G (*NCAPG*), ZW10 interactor (*ZWINT*), and denticleless E3 ubiquitin protein ligase homolog(*DTL)* are associated with poor OS of breast cancer patients. The enriched functions and pathways included cell cycle, oocyte meiosis and the p53 signaling pathway. The DEGs in breast cancer have the potential to become useful targets for the diagnosis and treatment of breast cancer.

## Introduction

Breast cancer has now overtaken lung cancer as the leading cause of cancer incidence worldwide, with an estimated 2.3 million new cases, accounting for 11.7% of all cancer cases^1^. In China, more than 300,000 women are diagnosed with breast cancer each year. About 70–80% of breast cancer patients with early stage non-metastatic disease can be cured, while advanced breast cancers with distant organ metastases are considered untreatable with currently available therapies^2^.

The global death rate from breast cancer is declining because of new therapeutic strategies, especially the targeted therapy. Increasing molecular targets have been discovered for breast cancer prognosis and therapy. In 2000, Perou and Sorlie reported that breast cancer could be divided into three subtypes according to the enrichment of three genes, luminal (estrogen receptor [ER]-positive), human epidermal growth factor receptor 2 (HER2, encoded by *ERBB2*)-positive and ER-negative, and basal subtypes^3^. Since then, several genes have been identified as predictive and prognostic biomarkers for breast cancer, which play important roles in targeted therapy. The most commonly used molecular-targeted drugs for HER2-positive breast cancer include tucatinib^4^, trastuzumab^5^, pertuzumab, lapatinib, neratinib and trastuzumab emtansine (T-DM1)^6,7^. Several drugs target the phosphoinositide 3-kinase (PI3K)/serine/threonine kinase(AKT) /mammalian target of rapamycin (mTOR) signaling pathway, including GDC-0068, Bez235, bupacoxib, abencoxib and alpelisib^8–10^. Vascular endothelial growth factor (VEGF) has also been identified as a key target for anti-angiogenic therapy, and its inhibitors bevacizumab, sorafenib, and sunitinib are also used for breast cancer therapy^11,12^. Androgen receptor (AR)-targeted therapies, including AR agonists and AR antagonists, have shown promising results in clinical trials for breast cancer patients, and combinations of AR-targeted therapies with other reagents (eg. PI3K inhibitor) have been studied to overcome resistance to AR-targeted therapies^13^. In addition, targeted therapies have been developed for epidermal growth factor receptor (EGFR), *BRCA1/2*-mutated polyadenosine diphosphate ribose polymerase (PARP), cyclin-dependent kinase 4/6 (CDK4/6), BTB and CNC homology1 (BACH1), and so on^14–18^. However, because of tumor heterogeneity, low ratios of responders, relapse and drug resistance, there is still an urgent need to identify new biomarkers that may aid the diagnosis and treatment of breast cancer.

Bioinformatics analysis is a valuable strategy for the comprehensive analysis of large databases, including complicated genetic information. In our study, we used sophisticated bioinformatics methods to screen potential biomarkers that may be useful for breast cancer. The Gene Expression Omnibus (GEO) [https://www.ncbi.nlm.nih.gov/geo/] database is an open database that allows researchers to select appropriate mRNA expression profiles. Online analysis tools are available to detect DEGs between tumors and normal tissues. In our study, we obtained three mRNA microarray datasets from the GEO (GSE134359, GSE31448, and GSE42568) and searched for DEGs using GEO2R. We then performed functional and pathway enrichment analyses of the identified DEGs using the DAVID database. PPI networks were constructed using STRING and visualized using Cytoscape. Conduct module analyses of the PPI network were performed using MCODE. The associations of these genes with OS were determined using an online Kaplan–Meier analysis tool. Finally, several breast cancer-related molecules were selected to investigate their potential role in a breast cancer diagnostic system.

## Methods

### Subjects and gene information

The GEO database is a national repository of genetic information databases, including microarray and next-generation sequencing data^19^. GEO2R is an online tool that can be used to detect DEGs from two or more GEO datasets. In this study, we retrieved three gene expression profiles (GSE134359, GSE31448 and GSE42568) from the GEO database. GSE134359 (Platform GPL17586) comprised 74 breast cancer samples and 12 noncancerous samples, GSE31448 (Platform GPL570) comprised 29 breast cancer samples and 4 noncancerous samples, and GSE42568 (Platform GPL570) comprised 104 breast cancer samples and 17 noncancerous samples. The characteristics of these datasets are shown in Table 1. More detailed patient information is provided in supplementary material 1. The “Reporting recommendations for tumor marker prognostic studies (REMARK)” were followed^20^.

**Table 1:** Summary of patient characteristics of three GEO datasets.

GEO accession	GSE134359	GSE31448	GSE42568
**Sample**			
Health	12	4	17
Tumor	74	29	104
**Histological type**			
Luminal A	24	1	NA
Luminal B	23	0	NA
HER2+	14	1	NA
Basal	13	25	NA

### Data analysis

We used GEO2R with screening criteria of adj. *P* < 0.05, log_2_ FC (fold change) > 1.5, or log_2_ FC <  − 1.5 to detect DEGs in breast cancer tissues compared with normal samples. We also used an online tool (http://bioinformatics.psb.ugent.be/webtools/Venn/) to plot Venn diagrams of the DEGs of three datasets.

### Gene ontology (GO) and Kyoto encyclopedia of genes and genomes (KEGG) pathway analysis

DAVID (http://david.ncifcrf.gov; version 6.8) is an open database that integrates biological data and analytical tools for functional annotation of genes and pathways^21^. GO is a bioinformatics tool for annotating genes and analyzing the biological processes they are involved in. KEGG is a database for analyzing relevant signaling pathways in largescale molecular datasets generated by high-throughput experimental techniques^22^. DAVID was used for GO enrichment analysis of the DEGs in terms of the molecular function, cell composition and biological process for each gene. KEGG pathway enrichment analysis was performed to clarify the function of the DEGs and the cell signaling pathways.

### PPI network visualization

We used the online analysis tool STRING (http://www.string-db.org/), with a confidence of 0.4, to construct the PPI network diagram for the identified DEGs. Cytoscape software^23^ was then used to construct the interaction network map, and the MCODE plug-in was used to screen the key gene modules in the network map. Cytoscape (version 3.8.0) is an open-source bioinformatics tool used to generate visual molecular interaction networks and the plug-in Molecular Complex Detection (MCODE) can develop key gene modules in the network. For this, we set the following parameters in MCODE: Degree Cut-off = 2, Node Score Cut-off = 0.2, K-Core = 2 and Max Depth = 100^24^.

### Kaplan–Meier survival and redundancy analyses of DEGs

A Kaplan–Meier plotter has been developed to evaluate the effects of 54,000 transcripts (mRNA, miRNA, protein) on survival for 21 types of cancer, including breast cancer (*n* = 6234), ovarian cancer (*n* = 2190), lung cancer (*n* = 3452), and stomach cancer (*n* = 1440)^25^. This database collates data from the GEO, European Genome-phenome Archive, and The Cancer Genome Atlas (TCGA) databases. The website was used to plot the OS for breast cancer patients for each gene. By selecting the best cutoff, a survival analysis was performed and False-Discovery Rate (FDR) was computed using the Benjamini–Hochberg method to correct for multiple hypothesis testing^26^. The hazard ratio (HR) and log-rank *P* values with 95% confidence intervals (CI) were calculated and displayed on the graph.

Cancer Dependency Map (Cancer DepMap, https://depmap.org/portal/), a RNAi and CRISPR-Cas9 knockout database^27,28^, was used to identified the functional target genes those are essential for breast cancer survival. The essential genes are potential therapeutic targets for breast cancer.

### Protein level verification

We visualized the selected hub-gene through ualcan^29^, and the protein expression data with 18 normal and 125 breast cancer samples were from CPTAC (Office of Cancer Clinical Proteomics Research, https://proteomics.cancer.gov/programs/cptac).

## Results

### Screening of DEGs

**Figure 1:**
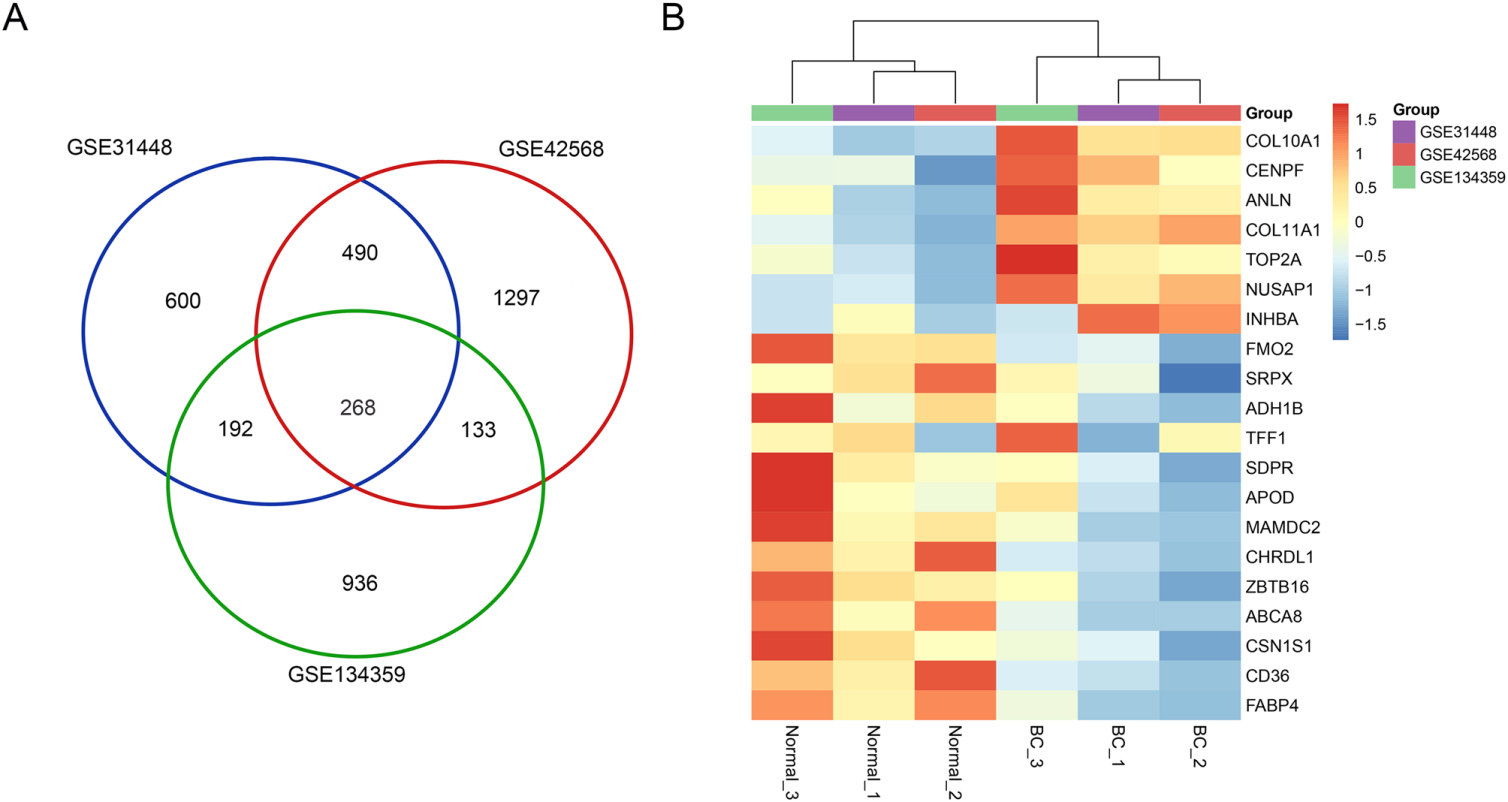
Identification of DEGs in the indicated breast cancer datasets. (**A**) Three online-available expression profiling datasets (GSE134359, GSE31448, GSE42568) were analyzed using GEO2R, and genes differentially expressed in breast tumor and peri-tumor samples (adj. *P* < 0.05 and |log_2_ FC |> 1.5) were defined as DEGs, followed by Venn diagram of DEGs. (**B**) Heatmap of top DEGs (adj.*P* < 0.05 and |log_2_ FC|> 3) in datasets GSE134359, GSE31448 and GSE42568.

A total of 1529, 1550, and 2188 DEGs were identified from the GSE134359, GSE31448, and GSE42568 datasets, respectively. Of these, 268 genes were present in all three datasets (Fig. 1A). 89 genes consistently showed high expression and 179 genes showed low expression in all three databases. The top 22 DEGs are shown on the heatmap, based on the criteria |log_2_ FC|> 3 and adj.*P* < 0.05 (Fig. 1B).

### GO and KEGG pathway enrichment analysis

**Figure 2:**
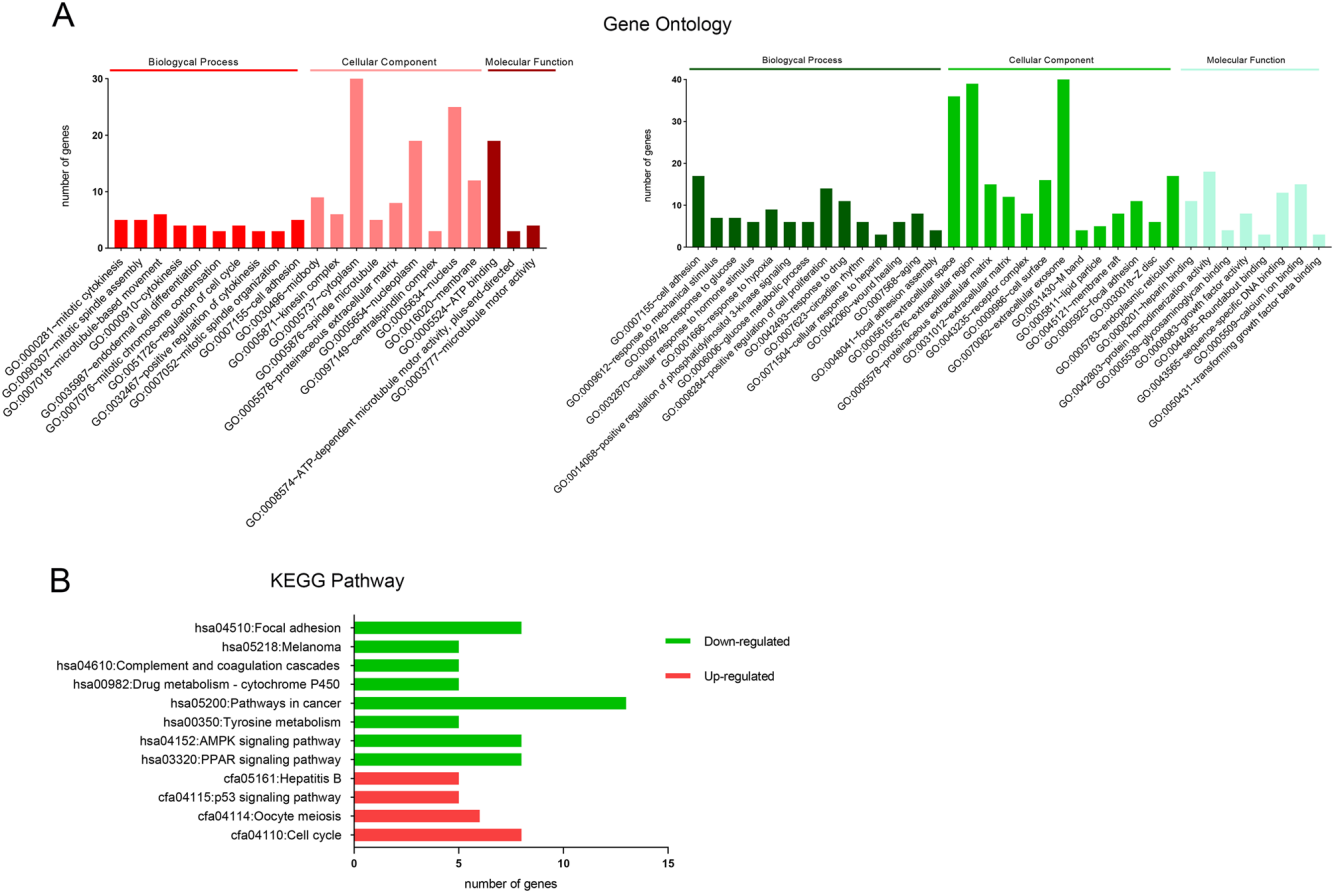
GO and KEGG analyses of DEGs. (**A**) GO analysis with up-regulated (red) and down-regulated (green) DEGs. Enriched GO items with P < 0.01 are shown, including biological process, cellular component, and molecular function. (**B**) KEGG analysis with up-regulated (red) and down-regulated (green) DEGs. Enriched KEGG pathways (P < 0.01) are shown.

GO enrichment and KEGG pathway analysis were performed on the DEGs using the DAVID database. GO enrichment analysis covers three aspects: biological processes, cell composition and molecular function (Fig. 2A). The upregulated genes were mainly related to mitotic cytokinesis, mitotic spindle assembly and microtubule-based movement; while the downregulated genes were mainly involved in cell adhesion, the response to mechanical stimuli and the response to glucose. The KEGG pathway analysis showed that the genes upregulated in tumors were enriched in cell cycle, oocyte meiosis and the P53 signaling pathway, while the downregulated genes were enriched in PPAR signaling pathway, AMPK signaling pathway, tyrosine metabolism, pathways in cancer and so on (Fig. 2B).

### PPI network construction and module selection

Considering the critical role of protein interactions in protein function, we used the STRING database and Cytoscape software to generate PPI network once we had identified the 268 DEGs. The results showed that there were dense regions in PPI, that is, genes closely related to breast cancer (HUB genes) modules.

**Figure 3:**
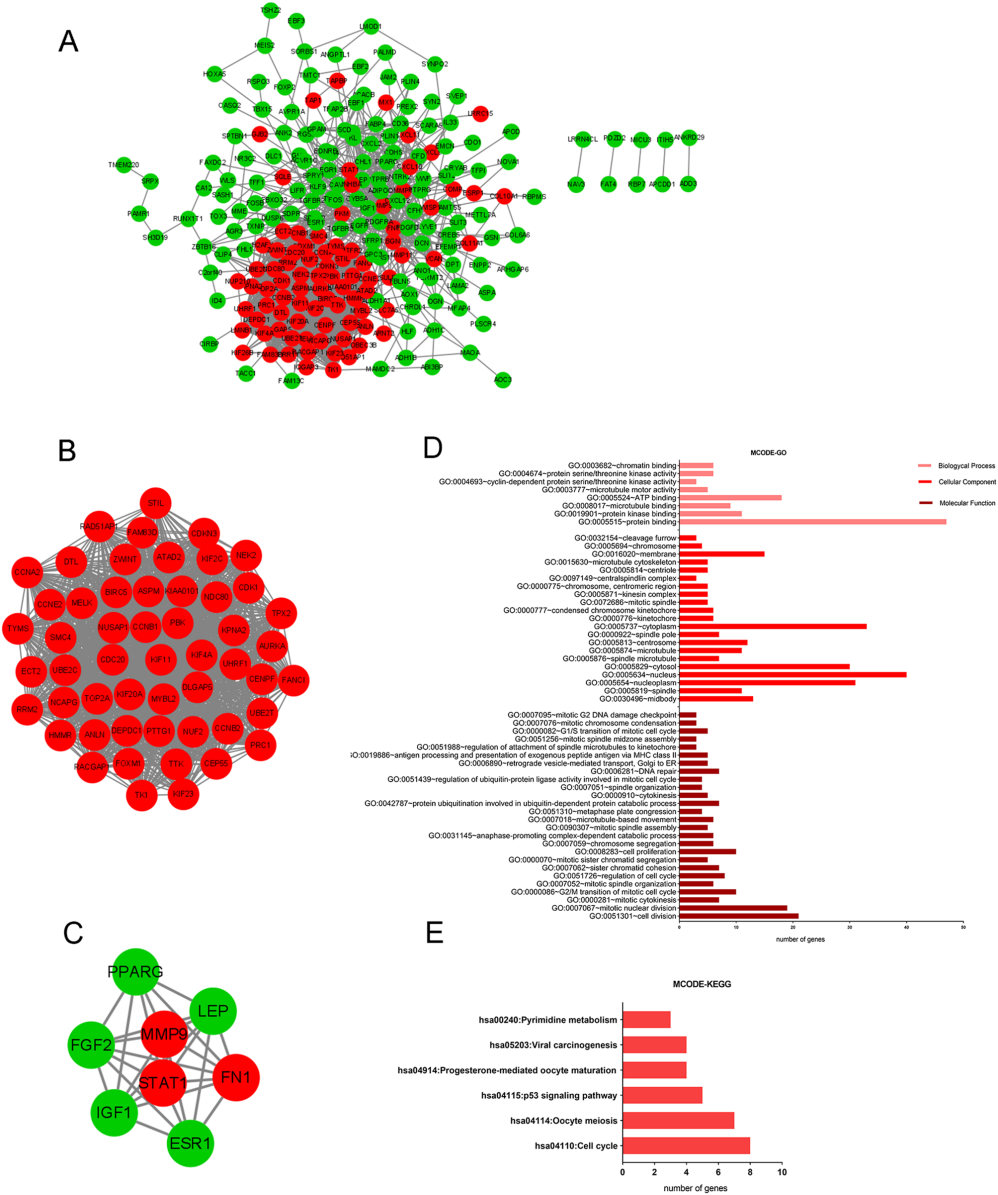
PPI and MCODE analyses of DEGs. (**A**) Protein–protein interaction network of 268 DEGs. (**B**) A significant module, containing 53 up-regulated proteins, was selected from protein–protein interaction network. (**C**) Another module selected from protein–protein interaction network. (D) GO analysis of MCODE genes. Enriched GO items with *P* < 0.01 are shown. (E) KEGG pathway analysis of MCODE genes. Enriched pathways with P < 0.05 are shown. For (**A**–**C**), red nodes are up-regulated proteins, and green nodes are down-regulated proteins. The lines represent the interaction relationship between nodes.

A total of 236 nodes and 2132 edges were selected to plot the PPI network, which consisted of 87 up-regulated genes and 149 down-regulated genes (Fig. 3A). Subsequently, a pivotal module of 53 genes (*CDK1*, *KIF11*, *DLGAP5*, *KIF4A* and so on) was identified with the degree ≥ 10 as the cut-off value by using MCODE (Fig. 3B). Another important module of 8 genes including both up-regulated and down-regulated genes was also identified (Fig. 3C). The top 10 HUB genes were identified by cytoHubba (Top 10 genes ranked in MCC). GO and KEGG analysis of these ten genes were conducted. HUB genes are related with cell division, mitotic cytokinesis in Biologycal Process; spindle, nucleus, spindle microtubule in Cellular Component; protein kinase binding, ATP binding in Molecular Function (Fig. 3D). They are also enriched in cell cycle, oocyte meiosis, p53 signaling pathway and so on (Fig. 3E).

### Survival and redundancy analyses

**Figure 4:**
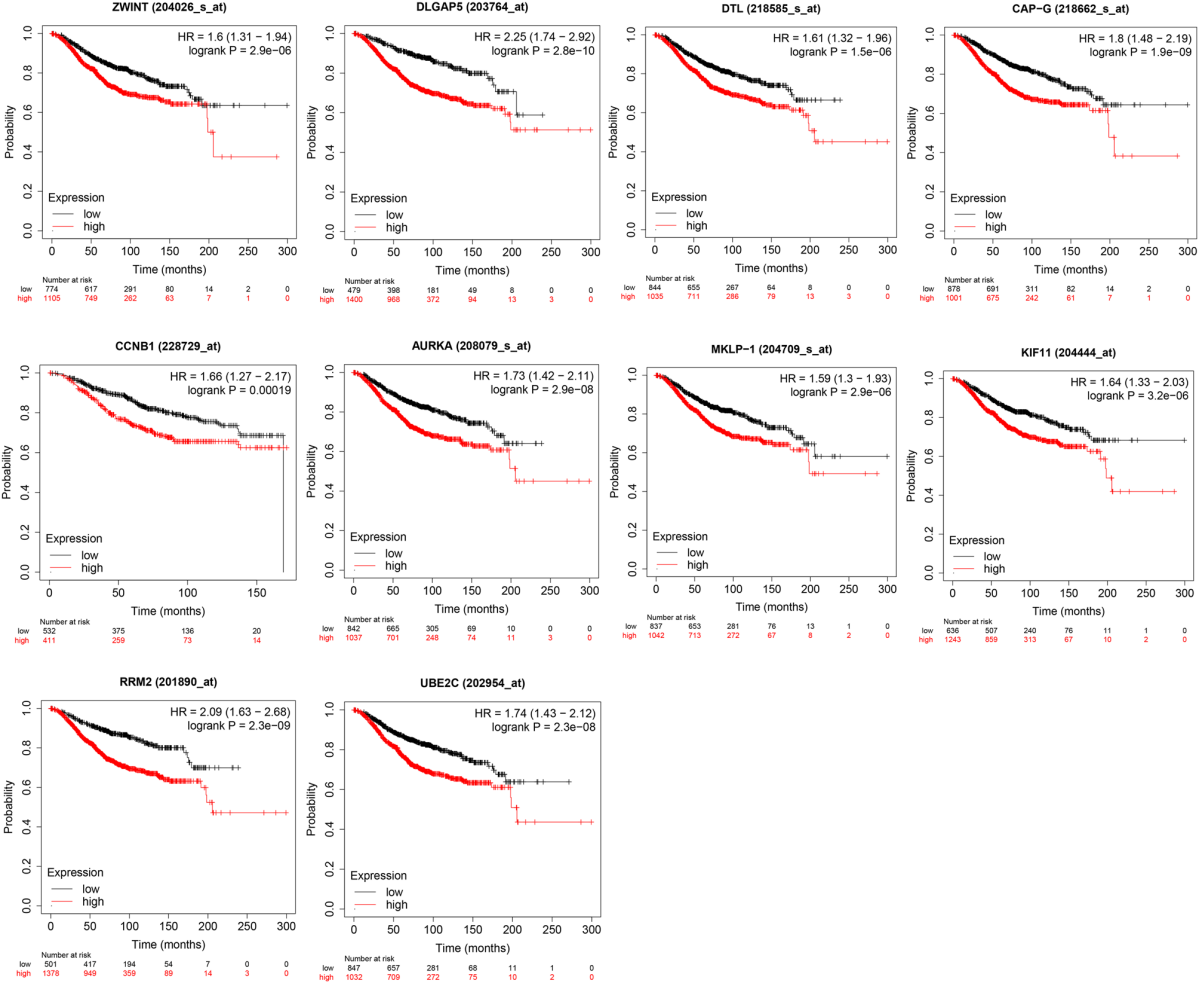
Prognostic estimation of the top 10 HUB genes. The top 10 HUB genes including ZWINT, DLGAP5, DTL, NCAPG, CCNB1, AURKA, KIF23, KIF11, RRM2 and UBE2C, were identified by cytoHubba, followed by survival analysis. Breast cancer patients were divided into two groups according to auto select best cutoff. Low, patients with gene expression lower than best cutoff; high, patients with gene expression higher than best cutoff.

Ten HUB genes in PPI network were evaluated for their prognostic value on the Kaplan–Meier plotter. All 10 genes exhibited their potential in the prediction of survival based on their expression. The OS for breast cancer patients was determined based on the expression level of each gene (low vs. high). As shown in Fig. 4, high mRNA expression of ZWINT (HR 1.6, 95% CI: 1.31–1.94, P < 2.9E−6, FDR 1%) was associated with a poorer OS for breast cancer patients, and this association also works for DLGAP5 (HR 2.25, 95% CI: 1.74–2.92, P = 2.8e−10, FDR 1%), DTL (HR 1.61, 95% CI: 1.32–1.96, P < 1.5E−6, FDR 1%), NCAPG(HR 1.6, 95% CI: 1.48–2.19, P < 1.9E−9, FDR 1%), CCNB1 (HR 1.66, 95% CI: 1.27–2.17, P < 0.00019, FDR 10%), AURKA (HR 1.73, 95% CI: 1.42–2.11, P < 2.9E−8, FDR 1%), KIF23 (HR 1.59, 95% CI: 1.3–1.93, P < 2.9E−6, FDR 1%), KIF11 (HR 1.64, 95% CI: 1.33–2.03, P < 3.2E−6, FDR 1%), RRM2 (HR 2.09, 95% CI: 1.63–2.68, P < 2.3E−9, FDR 1%) and UBE2C (HR 1.74, 95% CI: 1.43–2.12, P < 2.3E−8, FDR 1%). Among them, FDR of CCNB1 was 10%. Maybe the relationship between CCNB1 and survival is not obvious.

**Figure 5:**
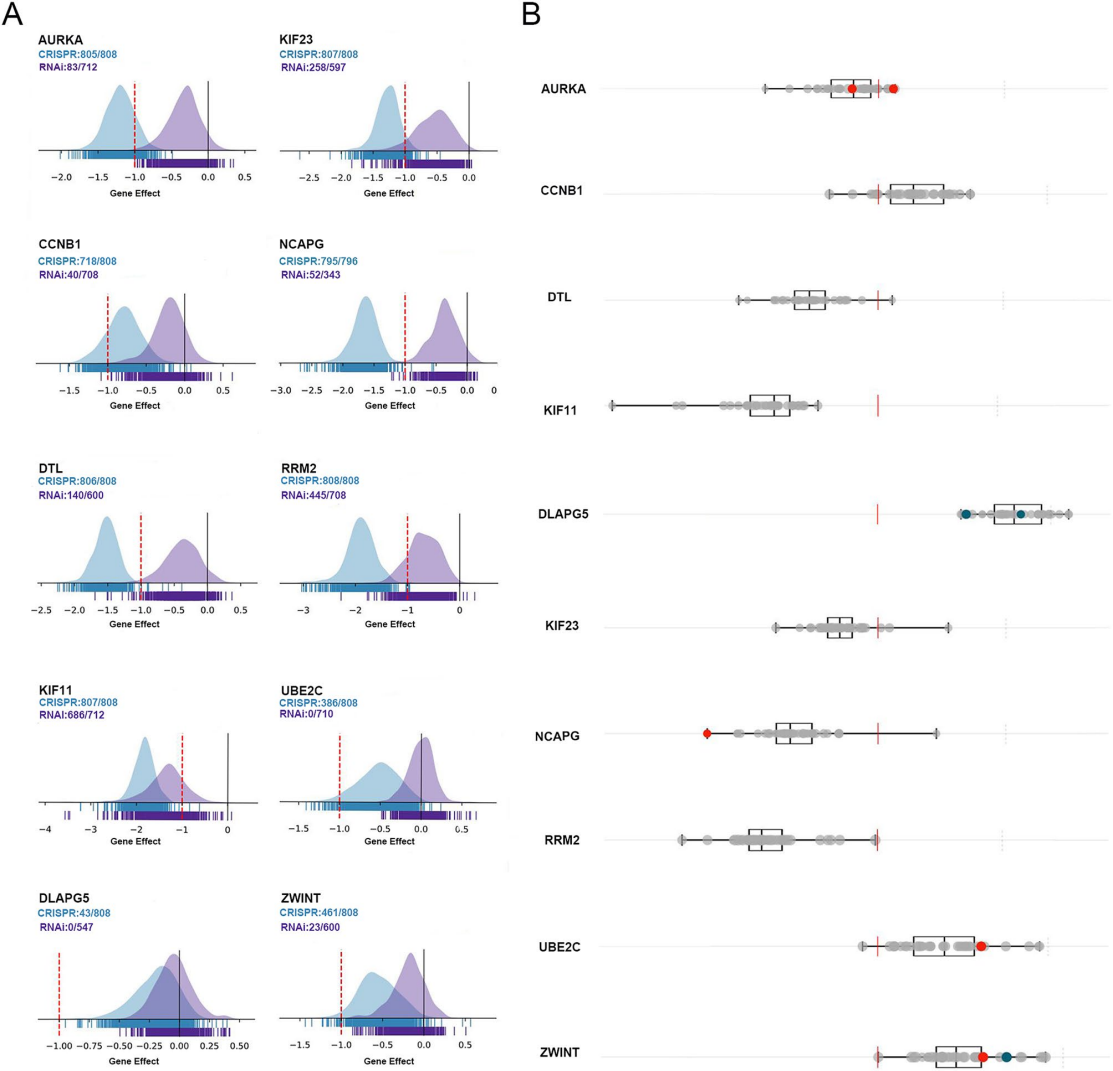
Redundancy analysis of the top 10 HUB genes. The essential role of indicated HUB genes in breast cancer cell survival was analyzed via DepMap, (https://depmap.org/portal/), which was established from CRISPR and RNAi screening data. (**A**) Redundancy analysis of ten genes in total cells lines. (**B**) The CERES dependency score of ten genes in breast cancer cells. A lower CERES score indicates a higher likelihood that the gene of interest is essential in a given cell line. A score of 0 indicates a gene is not essential (dotted line); − 1 is comparable to the median of all pan-essential genes (red line).

It is of great significance to analyze the role of HUB genes in breast cancer cell survival, and the essential genes are potential therapeutic targets. Here we analyzed the function of HUB genes using online-available DepMap tool, which was established based on CRISPR screening and siRNA screening data. There are 2 genes (*KIF11, RRM2*) that are common essential in both CRIAPR knockout and RNAi; 6 genes (*AURKA, CCNB1, DTL, KIF23, NCAPG, ZWINT*) that are common essential only in CRISPR knockout, indicating that these genes are not only diagnosis markers but also potential therapeutic targets (Fig. 5).

### Protein level verification

**Figure 6:**
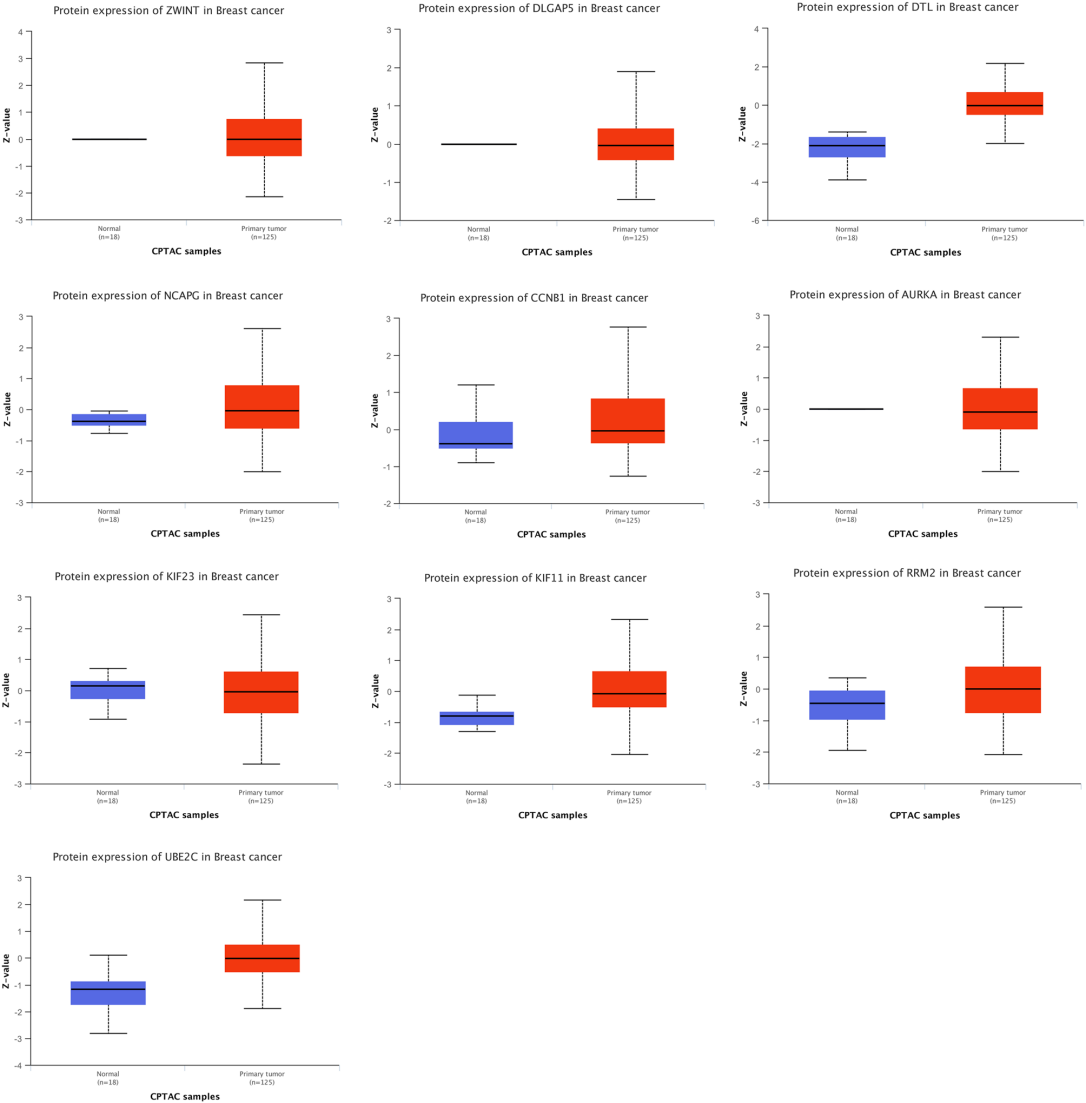
Protein expression of the top 10 HUB genes. The top 10 HUB genes, including ZWINT, DLGAP5, DTL, NCAPG, CCNB1, AURKA, KIF23, KIF11, RRM2 and UBE2C, were identified by cytoHubba, verified at the protein level by Ualcan. Z-values represent standard deviations from the median across samples for the given cancer type. Log2 Spectral count ratio values from CPTAC were first normalized within each sample profile, then normalized across samples.

Finally, we verified the screened HUB genes at the protein level (Fig. 6). The statistical significance of ZWINT (< 1E-12), DLGAP5 (< 1E−12), DTL (1.335246E-04), NCAPG (2.391440E−04), CCNB1 (3.434030E−03), AURKA (< 1E−12), KIF11 (5.4090702551948E−13), RRM2 (4.27904E−03) and UBE2C (1.99866742542919E−06) was less than 0.05, except KIF23 (9.61308686E−01).

## Discussion

Regardless of recent progress in the treatment of breast cancer, it has remained the most common cause of cancer-related deaths in the past few years. The high mortality rate of breast cancer is partly due to the lack of adequate screening methods with high sensitivity and specificity. Therefore, it is necessary to identify potential biomarkers for screening and early diagnosis of breast cancer. Microarray technologies and next-generation sequencing have become key tools for providing comprehensive genetic information on breast cancer samples and revealing the changes in disease progression. In this study, we used proven online bioinformatics tools to investigate possible biomarkers for diagnosis of breast cancer. We identified a total of 268 DEGs common to all three GEO datasets, which included 89 upregulated genes and 179 downregulated genes.

The upregulated genes were mainly involved in the three pathways, namely cell cycle, oocyte meiosis and the P53 signaling pathway, which are closely associated with cancer. The downregulated genes were mainly enriched in three other pathways: cell adhesion, the response to mechanical stimuli and the response to hormonal hypoxia. Among the identified DEGs, 87 showed high degrees in the PPI network. Further analysis revealed that the following 10 DEGs within these modules were closely associated with a shorter survival time of breast cancer patients: *DLGAP5*, *AURKA*, *UBE2C*, *CCNB1*, *RRM2*, *KIF23*, *KIF11*, *NCAPG*, *ZWINT* and *DTL*.

DLGAP5 is involved in Aurora A signaling and its neurogenic locus notch homolog protein 3 (NOTCH3) intracellular domain regulates transcription. DLGAP5 overexpression is associated with poor prognosis of breast cancer^30^. DLGAP5 is also associated with the prognosis of colorectal cancer, prostate cancer, and non-small cell lung cancer (NSCLC)^31–35^. A study identified a critical target of NOTCH3 signaling was the mitotic apparatus organizing protein DLGAP5 (HURP/DLG7)^36^. DLGAP5, which is regulated by nucleolar and spindle associated protein 1 (NUSAP1), is associated with the proliferation, migration and invasion of invasive breast cancer^37^. DLGAP5, required for AURKA-dependent, centrosome-independent mitotic spindle assembly, is essential for the survival and proliferation of *SMARCA4/BRG1* mutant^38^. One subpopulation of prostate cancerwas associated with enhanced expression of *DLGAP5* and decreased dependence upon androgen receptor signaling^39^.

AURKA plays an important role in cell cycle progression by promoting cell entry into mitosis, and is associated with increased risk of developing breast cancer. AURKA can translocate to the nucleus and enhance the phenotype of breast cancer stem cells, promoting unique oncogenic properties in malignant cells^40^. It has been reported that AURKA regulates the phenotype of breast cancer tumor stem cells by modifying and stabilizing Drosha mRNA with M6A^41^. In addition, AURKA plays an important role in the treatment of drug-resistant breast cancer^42^, and Aurora kinase A inhibitor has been in a five-arm phase 2 study for safety and activity^43^.

UBE2C can ubiquitinate Anaphase-Promoting Complex/Cyclosome (APC/C) (Ub)^44^. The high expression of UBE2C in breast cancer was reported to be an independent prognostic factor associated with increased risk of disease recurrence and death. Thus, it is considered as a potential therapeutic target for breast cancer^45–47^.

Cyclin B1, the protein encoded by *CCNB1*, is a regulatory protein involved in mitosis. It is necessary for proper control of the G2/M transition phase of the cell cycle. A study showed cyclin B1 and B2 transgenic mice are highly prone to tumors, including tumor types where B-type cyclins serve as prognosticators^48^. CCNB1 is associated with radiosensitivity in colorectal cancer^49^. CCNB1 can also affect cavernous sinus invasion in pituitary adenomas through the epithelial-mesenchymal transition^50^.

The gene *RRM2* encodes ribonucleotide reductase regulatory subunit M2, one of two non-identical subunits of ribonucleotide reductase. In a study that reported RRM2 acetylation at K95 suppresses tumor cell growth in vitro and in vivo, and is therefore a potentially attractive strategy for cancer therapy^51^. In a study that searched the GEO database for miRNA-mRNA or lncRNA-mRNA as novel biomarkers for breast cancer, the miR-21/RRM2 axis was identified as a candidate biomarker for the diagnosis and treatment of breast cancer^52^. In another study that showed a lincRNA, *lincNMR*, regulates tumor cell proliferation through a YBX1-RRM2-TYMS-TK1 axis governing nucleotide metabolism^53^. In addition, RRM2 was reported to be associated with the prognosis of prostate cancer^54^.

Kinesin family member 23, the protein encoded by *KIF23* is a member of the kinesin-like protein family, also known as MKLP1. MKLP1/KIF23 is the kinesin component of the centralspindlin complex^55^. It was reported that KIF23 expression is high in the majority of primary and metastatic lung cancer tissues or cell lines, and it is associated with poor survival^56^. In a study that examined the association between members of the kinesin family and breast cancer, KIF23 and KIF11 were found to be associated with poor prognosis^57^. KIF23 is regulated through wnt signaling pathway and associated with recurrence of hepatocellular carcinoma^58^.

Kinesin family member 11, the protein encoded by *KIF11*, is another member of the kinesin-like protein family. According to an Oncomine analysis of GEO and TCGA databases, KIF11 is a proto-oncogene associated with breast cancer and is significantly associated with poor prognosis^59^. KIF11 is also regulated through wnt signaling pathway and associated with recurrence of hepatocellular carcinoma.

NCAPG is a potential prognostic marker in HER2^+^ breast cancer, and a therapeutic target to effectively overcome trastuzumab resistance as well^60^. NCAPG has also been identified as a key gene in triple-negative breast cancer^61^ as well as hepatocellular carcinoma^62^. Furthermore, it was reported that high expression of NCAPG is associated with poor prognosis of various tumor types, and its overexpression may play an important role in the regulation of tumor-related pathways in tumor growth^63^.

Currently, little is known about the role of ZW10 interactor (ZWINT) in breast cancer. Denticleless E3 ubiquitin protein ligase homolog (DTL) is associated with proliferation and appears to be a promising molecular therapeutic target in breast cancer^64^. DTL may also be associated with poor prognosis of acral melanoma and gastric carcinoma^65,66^.

Based on redundancy analysis, two genes, *KIF11* and *RRM2,* may serve as therapeutic targets or prognostic indicators. The two genes are also differentially expressed by protein level verification. There are many differences between the predicted data and the clinical data, and the survival data derived from the Kaplan–Meier tool need to be validated. In future studies, more attention should be paid to breast cancer patients. There are many tumor subtypes for breast cancer, and it is necessary to define the biomarker characteristics of each subtypes. In our future study, we intend to recruit a cohort of breast cancer patients to investigate the sensitivity and specificity of these biomarkers for early screening of breast cancer; the results should facilitate the clinical application of these biomarkers for the diagnosis of breast cancer.

## Supplementary Information

Supplementary Information 1.

Supplementary Information 2.

## Data Availability

The datasets are available from the GEO database.
