# Supplementary material for: Screening and predicted value of potential biomarkers for breast cancer using bioinformatics analysis
Source: Sci Rep. 2021 Oct 21;11:20799. doi: 10.1038/s41598-021-00268-9 (PMC8531389; doi:10.1038/s41598-021-00268-9)
Supplement: Supplementary file 2 — Supplementary Information 2. [file 41598_2021_268_MOESM2_ESM.docx]

**Supplementary material 1. Sample information.** The table contains sample title and sample characteristics obtained from the GEO database.
